# *HiSPoD*: a program for high-speed polychromatic X-ray diffraction experiments and data analysis on polycrystalline samples

**DOI:** 10.1107/S1600577516005804

**Published:** 2016-06-17

**Authors:** Tao Sun, Kamel Fezzaa

**Affiliations:** a X-ray Science Division, Advanced Photon Source, Argonne National Laboratory, 9700 South Cass Avenue, Argonne, IL 60439, USA

**Keywords:** X-ray diffraction, high speed, polychromatic beam, dynamic processes

## Abstract

A high-speed X-ray diffraction technique was recently developed at the 32-ID-B beamline of the Advanced Photon Source for studying highly dynamic, yet non-repeatable and irreversible, materials processes. In experiments, the microstructure evolution in a single material event is probed by recording a series of diffraction patterns with extremely short exposure time and high frame rate. Owing to the limited flux in a short pulse and the polychromatic nature of the incident X-rays, analysis of the diffraction data is challenging. Here, *HiSPoD*, a stand-alone Matlab-based software for analyzing the polychromatic X-ray diffraction data from polycrystalline samples, is described. With *HiSPoD*, researchers are able to perform diffraction peak indexing, extraction of one-dimensional intensity profiles by integrating a two-dimensional diffraction pattern, and, more importantly, quantitative numerical simulations to obtain precise sample structure information.

## Abstract 2

Software to analyze polychromatic diffraction patterns from polycrystalline samples is described.

## 1. Introduction   

The emergence of the fourth-generation light source, the free-electron laser, did not mark the end of the ring-based synchrotron era. Owing to their complementary natures, free-electron lasers and synchrotron facilities will co-exist and serve the scientific community together for many years to come. One unique feature that ring-based synchrotrons possess is their time structure. The high repetition rate of the photon pulses enables researchers to study many transient phenomena which cannot be studied using a free-electron laser, including (i) dynamic structural evolution of fluids and particle suspensions in processes such as fuel spray, drop impact, shear thickening, *etc.* (Wang *et al.*, 2008 ▸; Moon *et al.*, 2014 ▸; Fezzaa & Wang, 2008 ▸; Lee *et al.*, 2011 ▸); (ii) behaviors and failures of materials under extreme conditions, such as high-rate (high-pressure) loading, thermal shocking, *etc.* (Jensen *et al.*, 2012 ▸; Hudspeth *et al.*, 2013 ▸; Luo *et al.*, 2012 ▸; Parab *et al.*, 2014 ▸); and (iii) rapid materials processing and machining, such as laser processing, combustion synthesis, casting, welding, *etc.* (Barron *et al.*, 2013 ▸; Sullivan *et al.*, 2012 ▸; Reeves *et al.*, 2009 ▸). These processes are highly dynamic, yet non-repeatable and/or irreversible, and therefore they cannot be probed using conventional pump–probe techniques.

The 32-ID-B beamline at the Advanced Photon Source (APS) of Argonne National Laboratory, USA, is dedicated to high-speed X-ray experiments using undulator white or pink beams. By taking advantage of the time structure of the filling pattern in the storage ring, single-pulse full-field X-ray phase-contrast images can be obtained with an exposure time set by the natural width of the pulse (down to 80 ps) and a frame rate set by the bunch repetition frequency (up to 6.5 MHz) (Jensen *et al.*, 2015 ▸). Recently, the high-speed diffraction technique was developed and implemented at the beamline. Users now can record high-speed movies of a single material event in both real and reciprocal spaces (Fan *et al.*, 2014 ▸; Hudspeth *et al.*, 2015 ▸). Compared with conventional monochromatic beam diffraction, white-beam diffraction data from polycrystalline samples are generally more challenging to analyze, as will be elaborated in the following section. To address this issue, we developed a stand-alone Matlab® software *HiSPoD* (for High Speed Polychromatic Diffraction) to facilitate user data analysis.

## 2. High-speed white-beam diffraction   

**Figure 1:**
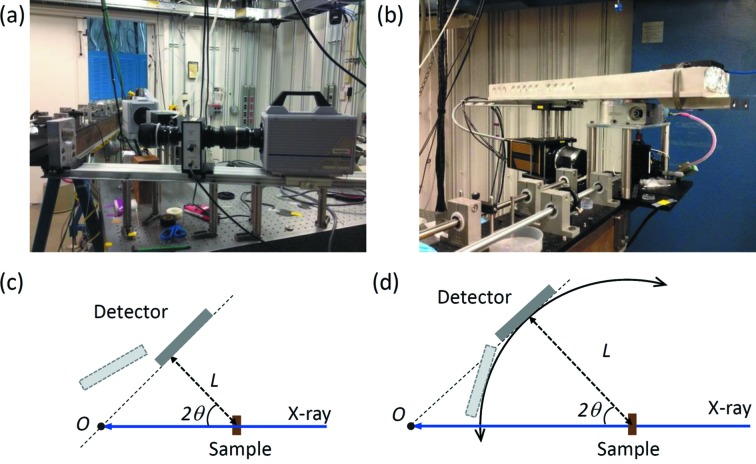
Geometries of high-speed diffraction experiments. (*a*, *b*) Photographs showing the detection system and mounting options. (*c*, *d*) Schematic of detector positioning in either the arbitrary mode or circular track mode, corresponding to the cases shown in (*a*) and (*b*), respectively.

The high-speed diffraction instrument in the 32-ID-B beamline of the APS has been reported previously (Fan *et al.*, 2014 ▸; Hudspeth *et al.*, 2015 ▸). In brief, the detection systems we built consist of three basic components: a scintillator that converts the X-ray intensity to visible-light signal; an image intensifier that amplifies the low-light-level images; and a visible-light area detector to record diffraction patterns. The detection system can be either mounted directly on an optics table (Fig. 1*a* ▸) or on a motorized rotation arm (Fig. 1*b* ▸) if frequent adjustment of the detection angle is needed during the experiment. *HiSPoD* is able to deal with both detector geometries (Figs. 1*c* and 1*d* ▸), as long as some position parameters are measured or calibrated.

**Figure 2:**
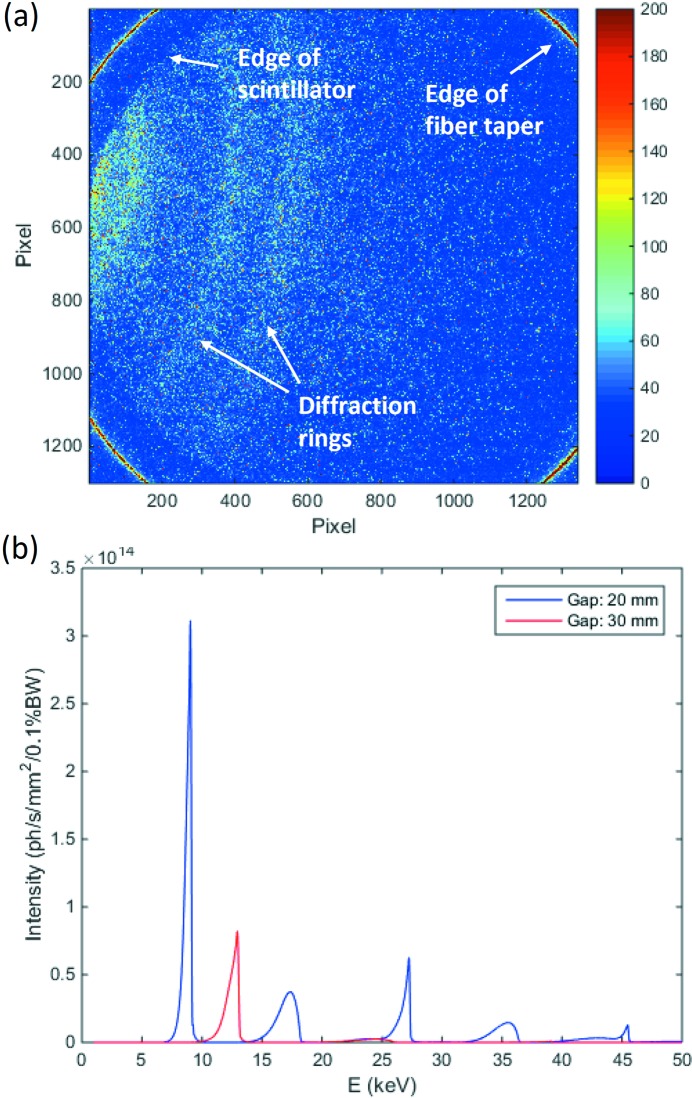
High-speed diffraction pattern and energy spectra of white beams. (*a*) A typical single-pulse white-beam X-ray diffraction pattern from an aluminium sample. (*b*) Energy spectra of X-rays generated by undulator A (3.3 cm) with 20 mm and 30 mm gaps.

The challenges in analyzing white-beam diffraction data stem from two factors. First, due to the limited flux in a short X-ray pulse and the intrinsic limitation of the detection system, the signal-to-noise ratio of the diffraction pattern from a polycrystalline sample is relatively low. Fig. 2(*a*) ▸ shows a single-pulse diffraction pattern from an aluminium sample, collected using an intensified charge-coupled device (ICCD; Princeton Instruments PI-MAX). A LYSO scintillator (50.8 mm diameter, 250 µm thickness) is coupled to the ICCD using a fiber taper (2:1 ratio). The gap of the undulator (Type A, 3.3 cm period) was set to 30 mm, which generates a flux of ∼7 × 10^8^ photons s^−1^ mm^−2^ (0.1% bandwidth)^−1^ in a single pulse. In the diffraction pattern, the intense ring-shape feature appearing at the corners is the edge of the taper, and the edge of the scintillator can be also observed as marked in Fig. 2(*a*) ▸. The diffraction rings are barely discernable though due to the high level of noise. Second, a white beam generated by an undulator contains X-rays with energies covering the entire range. Different from a beam generated from bending magnets whose energy spectrum is mostly continuous, a typical spectrum of an undulator white beam contains multiple harmonic peaks, each of which exhibits a few percentage in bandwidth. Fig. 2(*b*) ▸ shows the energy spectra of the X-ray beam generated by undulator A (3.3 cm) with 20 mm and 30 mm gaps, and the first harmonic energies are about 9 keV and 13 keV, respectively. For a polycrystalline sample, its diffraction peaks from multiple X-ray harmonic energies may co-exist and even overlap in a diffraction pattern. Therefore, identifying the diffraction peaks and quantifying sample structure information become challenging.

## 3. Data analysis with *HiSPoD*   

**Figure 3:**
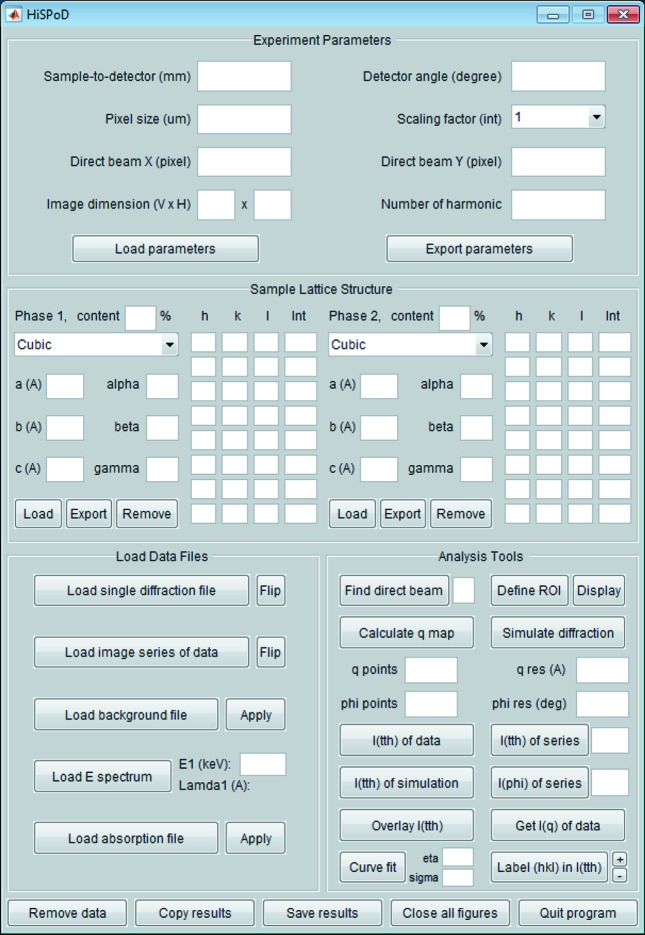
*HiSPoD* user interface.

*HiSPoD* is a GUI-assisted software that runs in Matlab®. *Image Processing Toolbox* is required for using some of the functions in this program. Fig. 3 ▸ shows the user interface of *HiSPoD*. The major functions are grouped into five modules.

(i) In the ‘Experiment Parameters’ module, one can input/load/save basic parameters associated with the detector and experiment geometry. Among these parameters, ‘Pixel size’ and ‘Image dimension’ are unambiguous; ‘Sample-to-detector’ and ‘Detector angle’ can be measured roughly in the experiment and calibrated using diffraction data from reference samples; ‘Direct beam X’ and ‘Direct beam Y’ can be obtained using the software by analyzing reference diffraction patterns.

(ii) In the ‘Sample Lattice Structures’ module, one can input/load/save sample lattice parameters, and reference diffraction information. Such information can be obtained from the International Centre for Diffraction Data, in-house diffraction experiments, or commercial software, such as *CrystalMaker*. Structure information of two phases can be input for analysis.

(iii) In the ‘Load Files’ module, one can load a single diffraction pattern or a data series, background file, energy spectrum and sample/filter absorption file.

(iv) In the ‘Analysis Tools’ module, multiple analysis tools for a variety of data analysis are available, which will be described in detail in the following sections.

(v) At the bottom of the user interface, a few system tools are available, allowing users to remove, copy and save data and analysis results.

### 3.1. Major functions for data analysis   

#### 3.1.1. Extract one-dimensional intensity profiles from two-dimensional diffraction patterns   

**Figure 4:**
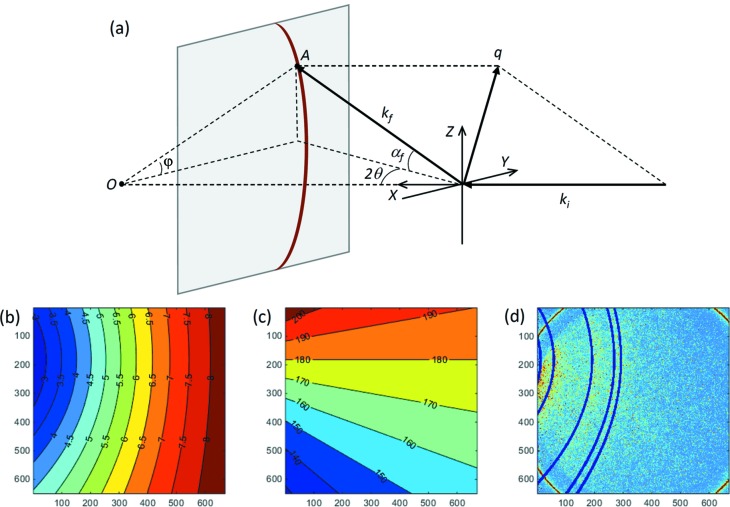
Calculation of *q* and φ maps. (*a*) Schematic of the scattering geometry. (*b*) Map of scattering vector *q* (first-harmonic). (*c*) Map of azimuthal scattering angle φ. (*d*) A typical diffraction pattern, in which the reference diffraction peaks (first-harmonic) are superimposed.

When dealing with noisy data, obtaining a one-dimensional (1D) diffraction intensity profile by integrating a two-dimensional (2D) pattern could be the first data treatment one intends to perform before further analysis. *HiSPoD* provides users with tools for obtaining diffraction intensities as functions of scattering vector *q* and azimuthal angle φ. Fig. 4(*a*) ▸ shows the scattering geometry. *k*_i_ and *k*_f_ are wavevectors of the incident and diffracted beams, respectively, as 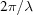 (λ is the X-ray wavelength). For a point A on the detector plane, the scattering vector *q* can be calculated as
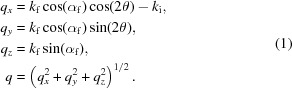
For a polycrystalline sample, the diffraction peaks appear at discrete *q* positions (*i.e.* 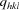 = 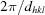; *d*_*hkl*_ is the spacing of the *hkl* atomic plane). In the case of an undulator white beam, diffraction peaks corresponding to different photon harmonic energies may overlap in the detectable reciprocal space. Once the experiment parameters, the sample lattice structure and the energy spectrum are loaded into *HiSPoD*, one can calculate the *q* map (corresponding to the first-harmonic energy) and φ map, as shown in Figs. 4(*b*) and 4(*c*) ▸. In the meantime, if the experiment data are also loaded, the diffraction peak positions (first-harmonic energy only) will be superimposed onto the diffraction pattern to facilitate the optimization of the experiment parameters, as shown in Fig. 4(*d*) ▸.

**Figure 5:**
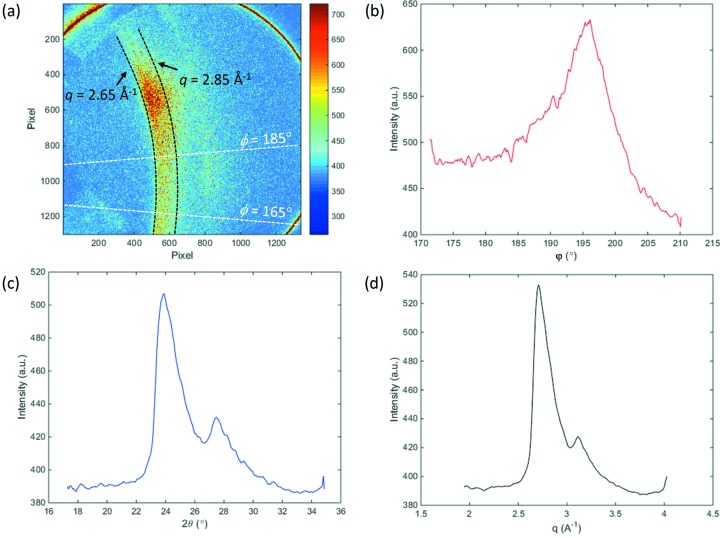
Extraction of 1D intensity profiles from a 2D diffraction pattern. (*a*) Diffraction pattern from a textured aluminium sample (undulator gap 30 mm, exposure time 3.37 µs). (*b*) Diffraction intensity as a function of azimuthal angle φ, obtained by integrating over the *q* range 2.65–2.85 Å^−1^. (*c*) Diffraction intensity as a function of scattering angle 2θ, obtained by integrating over the φ range 165–185°. (*d*) Diffraction intensity as a function of first-harmonic scattering vector *q*, obtained by integrating over the entire φ range.

Fig. 5 ▸ shows different 1D diffraction intensities one can obtain using *HiSPoD*. The diffraction pattern (Fig. 5*a* ▸) from another aluminium sample is chosen here in order to show a plot of *I*(φ) with some features. Fig. 5(*b*) ▸ shows the diffraction intensity distribution across the azimuthal angle φ, integrated over the defined *q* range of 2.65–2.85 Å^−1^. A peak around φ = 196° in the plot can be readily observed, indicating a texture structure of the sample. Fig. 5(*c*) ▸ shows the diffraction intensity as a function of the scattering angle, integrated over the defined φ range of 165–185°. The broadening of the diffraction peak is mostly due to the bandwidth of the photon energies, though fine crystal size, potential lattice distortion, point spread function of the detection system, and overlapping of diffraction peaks from different harmonic energies all contribute to the peak broadening. In addition, one can also plot the diffraction intensity as a function of a chosen scattering vector *q*. Fig. 5(*d*) ▸ shows the case of the *q* vector corresponding to the first-harmonic energy. This diffraction intensity is averaged over the entire φ range. Although researchers tend to have more intuitive sense of a *I*(*q*) plot, one should pay extra attention when trying to estimate sample *d*-spacings from *I*(*q*) peak positions. As previously mentioned, diffraction peaks of different atomic planes from different harmonic energies may appear at the same scattering angle.

#### 3.1.2. Index diffraction peaks   

**Figure 6:**
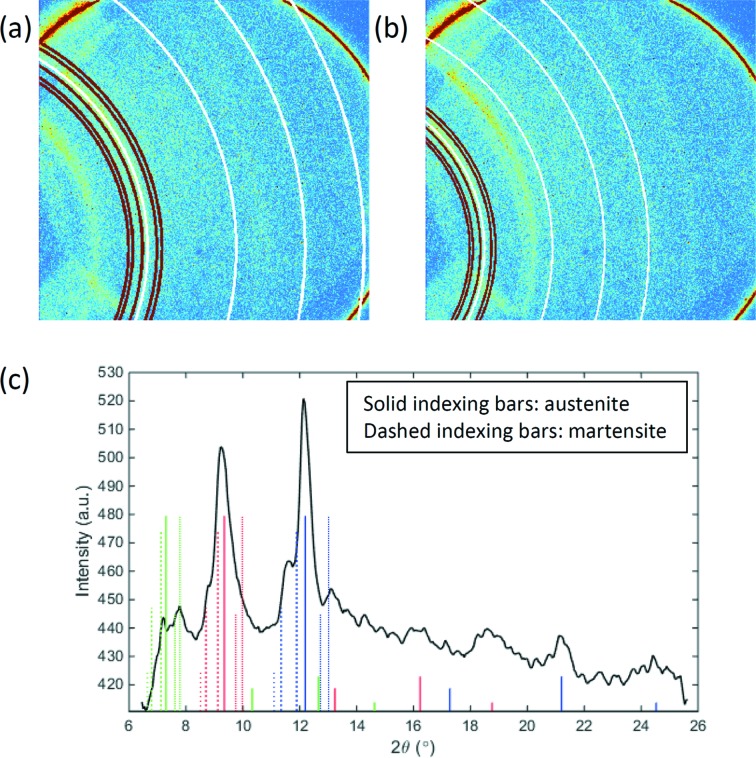
Diffraction peak indexing using *HiSPoD*. (*a*, *b*) Indexing of a diffraction pattern from a NiTi sample, collected during a high-rate tensile loading (undulator gap 20 mm, exposure time 3.37 µs). The reference diffraction peaks for austenite (white) and martensite (red) phases are superimposed. Cases are shown of the third-harmonic (*a*) and fourth-harmonic (*b*) energies. (*c*) Indexing of 1D diffraction intensity. Blue, red and green indexing bars correspond to the third-, fourth- and fifth-harmonic energies, respectively. Solid indexing bars are for the austenite phase, and dotted bars are for the martensite.

To help beamline users understand their diffraction data properly, *HiSPoD* offers functions for indexing both 2D and 1D diffraction data. 2D peak indexing has been briefly described above, and an example is shown in Fig. 4(*d*) ▸. Here, a case of a more complex sample, NiTi, is illustrated in Fig. 6 ▸. In the experiment, the NiTi sample was subjected to a high-rate tensile loading using a Kolsky bar system. The gap of the undulator was set to 20 mm. The diffraction pattern was collected in the middle of the rapid tensile pulling process with an exposure time of 3.37 µs. Figs. 6(*a*) and 6(*b*) ▸ show the same diffraction pattern superimposed with indexing rings corresponding to the third- and fourth-harmonic energies, respectively. In each figure, the white rings mark the peak positions of the austenite phase, while the red rings marked the martensite peak positions. Fig. 6(*c*) ▸ shows the peak indexing for 1D diffraction intensity profile, in which different colors of the indexing lines represent different harmonic energies and the line style distinguishes two phases. This particular diffraction pattern indicates the co-existence of austenite and martensite phases in the sample during the stress-induced phase transformation process.

#### 3.1.3. Quantitative data simulation   

In order to obtain *d*-spacings of the sample, *HiSPoD* provides the function for quantitative simulation of the diffraction data. Since most user experiments use the transmission geometry, the original energy spectrum needs to be modified due to sample absorption. This is particularly important when simulating diffraction data with peaks from different harmonic energies. The simulation of a white-beam diffraction pattern from a known material starts from the calculation of monochromatic beam diffraction patterns for the specific detector location, 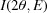. Then these mono-beam diffraction patterns are integrated over the entire energy range with weighting factor being the flux of photons with different energy, 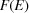,
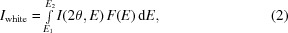
where *E*_1_ and *E*_2_ are typically 1 keV and 60 keV, respectively. The values of *E*_1_ and *E*_2_ are flexible and could be specifically defined when loading the energy spectrum file. For the undulator conditions generally used, the first-harmonic energy is above 5 keV, and the integrated flux of photons with energy higher than 60 keV is less than 1% of the flux of the photons with lower energies. Another factor one may consider when selecting *E*_1_ and *E*_2_ values is the scattering angle. Owing to the limited angle range that the detector covers, diffraction generated by photons with very low energy (giving high diffraction angles) or very high energy (giving low diffraction angles) could be neglected.

**Figure 7:**
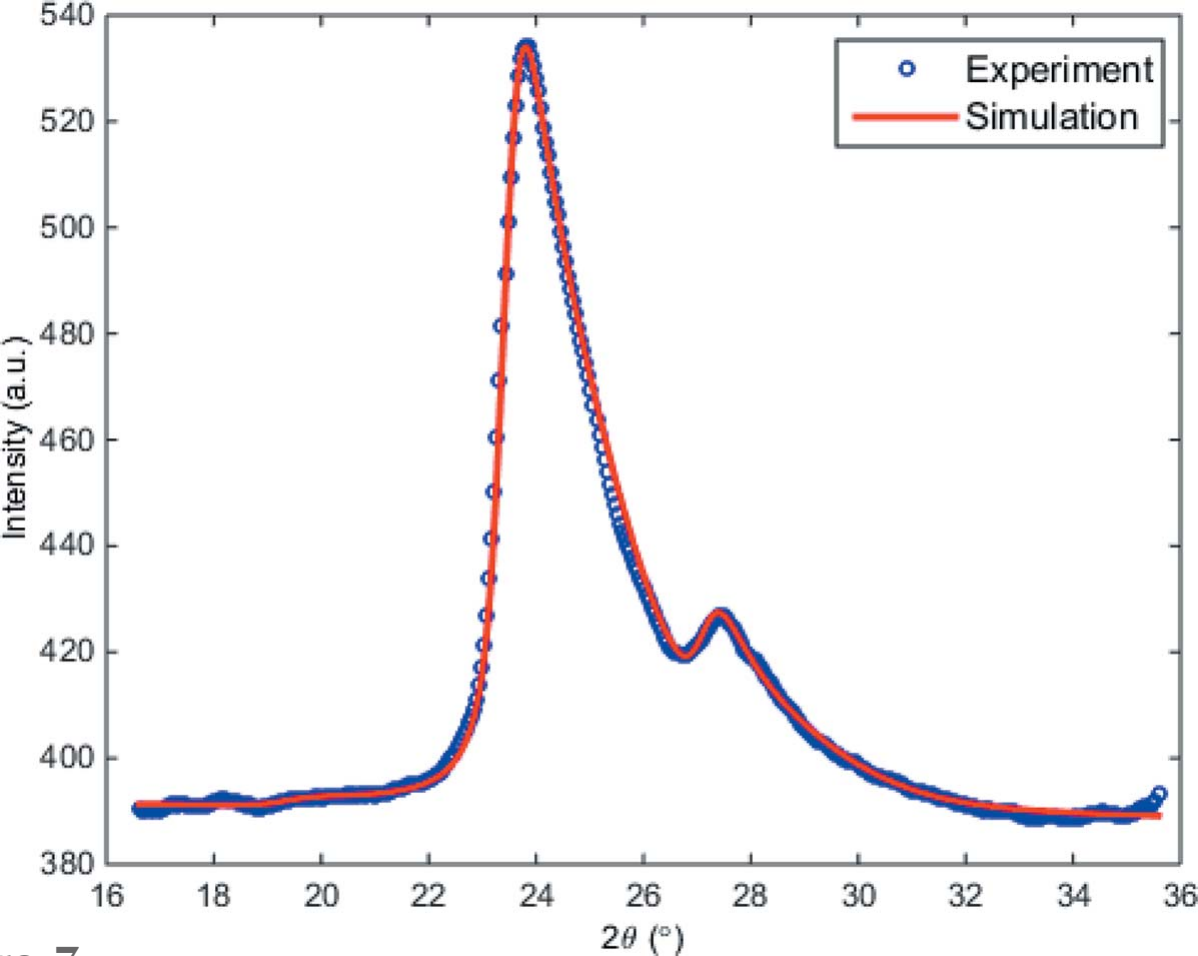
1D diffraction intensity (extracted from the 2D pattern shown in Fig. 5*a* ▸) and corresponding simulation.

To improve the calculation speed, discrete diffraction peaks are considered, meaning that 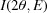 is normally replaced by a series of 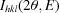. Equation (2) then becomes
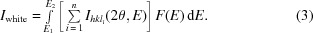
Here, the diffraction peak shape is described using the pseudo-Voigt function
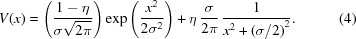
Essentially, the white-beam diffraction intensity at a given scattering angle is the convolution of the input diffraction intensities of different atomic planes with the sample-absorption-modified energy spectrum of the incident X-rays. Fig. 7 ▸ shows the numerical simulation and experiment diffraction data of the aluminium sample, previously described in Fig. 5 ▸. The quantitative agreement between data and simulation can be well observed.

### 3.2. Other features   

#### 3.2.1. Estimate direct beam position   

**Figure 8:**
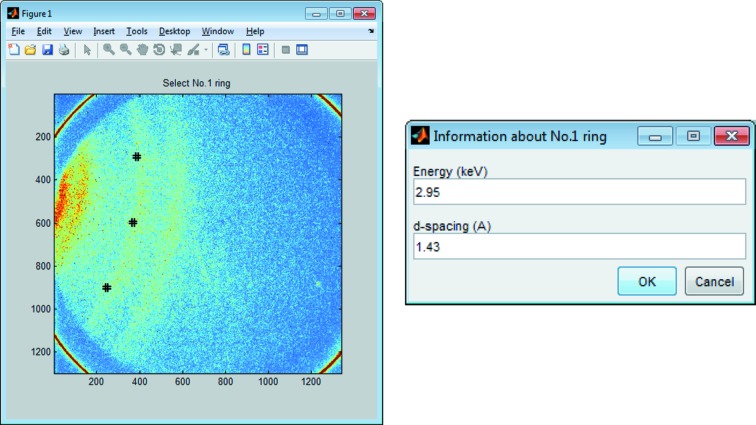
Screenshots illustrating the ‘Find Direct Beam’ function.

Most of the parameters associated with the detector position in *HiSPoD* can be measured in the experiment with a precision that is sufficient as the initial guess. However, finding the direct beam position could be tricky. Since simultaneous imaging and diffraction is performed in a typical user experiment, the transmitted beam falls on the imaging detector, but not the diffraction detector, as illustrated in Fig. 1(*c*) ▸. If the diffraction detection system is mounted on a diffractometer and the sample is at the rotation center, the estimation of direct beam *X* and *Y* positions is relatively straightforward. However, often the user instruments for triggering the sample event largely constrain the space for mounting and rotating the detector freely. Therefore, in such experiments, the detector is mounted directly on the optical table (Figs. 1*a* and 1*c* ▸), and a thorough alignment of the detector position is restricted. In *HiSPoD*, a function called ‘Find Direct Beam’ was developed to help users quickly estimate the direct beam position. As shown in Fig. 8 ▸, in a diffraction pattern from a reference sample, one may click a few points to select a diffraction ring, and input the associated sample *d*-spacing and X-ray harmonic energy. The software will calculate the direct beam position and automatically input the numbers of ‘Direct beam X’ and ‘Direct beam Y’ into the ‘Experiment Parameters’ module. This result serves as the initial estimation; one may further calibrate the parameters by running quantitative simulation to match the diffraction data from reference samples.

#### 3.2.2. Define region of interest   

**Figure 9:**
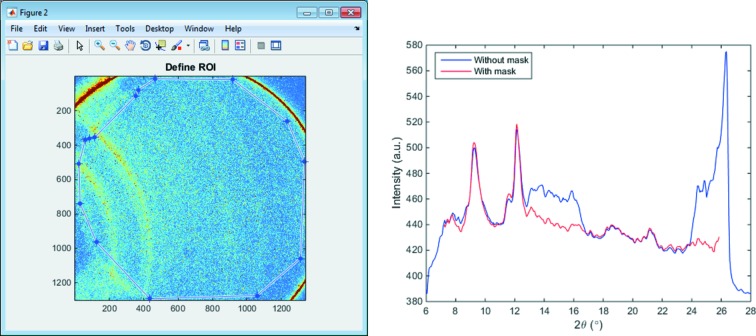
Definition of region-of-interest, and its effect on extracting the 1D diffraction intensity.

When extracting the 1D intensity profile from a 2D diffraction pattern, *HiSPoD* gives users the flexibility to mask out any shape of a region-of-interest (ROI). As briefly described in Fig. 2 ▸, with current detection systems, many bright features on a diffraction pattern are not sample diffraction signals. As the signal-to-noise ratio of a high-speed diffraction pattern is relatively low, a careful definition of the ROI becomes important. Fig. 9 ▸ shows the difference in 1D intensity profiles with and without selecting an integration mask.

#### 3.2.3. Batch analysis   

**Figure 10:**
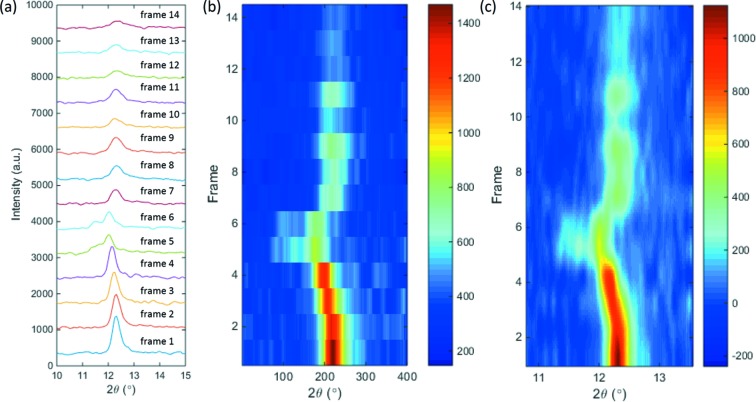
Batch analysis results of a NiTi sample subjected to high-rate tensile loading (undulator gap 20 mm, frame rate 20 kHz, exposure time 5 µs). (*a*) 1D diffraction intensity stack-up. (*b*) 2D graph showing the diffraction intensity change as a function of frame number (or time delay). (*c*) Smoothed 2D intensity graph providing a better visualization effect.

Batch data analysis is another convenient feature that *HiSPoD* offers. In high-speed diffraction experiments at beamline 32-ID-B, a high-frame-rate movie with a series of 2D diffraction patterns is recorded for each sample event routinely. To facilitate the analysis, *HiSPoD* allows one to load the data series as a batch and calculate all 1D intensity profiles (*i.e.* *I*–*q* or *I*–φ) by clicking one button. The outcome will be displayed in two formats, as shown in Fig. 10 ▸. Fig. 10(*a*) ▸ piles up the 1D profiles vertically by plotting them in their original curve format, while Fig. 10(*b*) ▸ organizes them together into a 2D graph with the horizontal axis as the scattering angle, vertical axis as the frame number (or time delay), and the color map indicating the diffraction intensity. Fig. 10(*c*) ▸ is the smoothed and high-resolution version of Fig. 10(*b*) ▸, obtained by data interpolation, and it is displayed to promote the visualization of the sample structure evolution.

#### 3.2.4. User-friendly pop-up messages   

Besides all the data analysis functions, the design of *HiSPoD* tries to be concise, intuitive and user friendly. When using a function in the ‘Analysis Tools’ module, a message window will pop-up with step-by-step instructions for users to follow. Also, if a mis­operation occurs or certain parameters are missing, *HiSPoD* will deliver a specific error message showing what is wrong, so that users without experience in Matlab® programming will feel comfortable using this software.

## 4. Summary and outlook   

*HiSPoD* provides users with an effective tool for (i) designing experiments by predicting sample diffraction patterns before the visit to the synchrotron facility, (ii) optimizing parameters and adjusting on-site work plan by quickly analyzing the experiment data, and (iii) performing off-site analysis and quantitative simulations to fully understand the data. With the increasing diversity of the beamline experiments, more functions will be added to *HiSPoD* to serve the specific need for different user experiments. Meanwhile, users are also welcome to contribute their home-built functions. *HiSPoD* is developed for analyzing undulator white-beam diffraction data from polycrystalline samples. For single-crystalline samples, diffraction using a white beam is often termed Laue diffraction. In addition to the detector geometry, the analysis software needs to take the sample crystallographic orientation into account for indexing the diffraction spots and quantifying the strain. With much software available now for conventional Laue diffraction data analysis, we do not intend to add similar capability to *HiSPoD*, but will consider so upon increasing user demand. Nevertheless, in the experiment, if the beam is incident on a single-crystalline sample at the Bragg angle, *HiSPoD* in its present version will be able to analyze the diffraction spot to extract information associated with peak broadening and lattice strain. *HiSPoD* is currently distributed to users of beamline 32-ID-B at the APS. With the white/pink beam high-speed diffraction technique being implemented at many other synchrotron facilities (Eakins & Chapman, 2014 ▸; Lambert *et al.*, 2014 ▸; Rack *et al.*, 2016 ▸), we believe *HiSPoD* will find broader applications in studies of highly dynamic material processes. High-speed experiments using monochromatic beam remains challenging at a synchrotron due to the limited flux. However, with an upgraded source (*i.e.* diffraction-limited storage rings), the implementation of advanced insertion devices (*i.e.* superconductor undulators), and the much improved detectors (*i.e.* direct-detection multi-frame high-speed detectors), high-speed X-ray experiments using high-energy monochromatic beam will become feasible in the foreseeable future.
